# Estimation of Reference Values of Gait Spatiotemporal and Kinematic Parameters in the Lower Extremities and Trunk Using a Markerless Motion Capture System for Healthy Older Japanese Adults

**DOI:** 10.1298/ptr.E10247

**Published:** 2023-09-08

**Authors:** Hungu JUNG, Shunsuke YAMASHINA, Ryo YAMASAKI, Yu INOUE, Kazuaki HAMADA, Kenta HIROHAMA, Shigeharu TANAKA, Ryo TANAKA

**Affiliations:** ^1^Graduate School of Humanities and Social Sciences, Hiroshima University, Japan

**Keywords:** Gait, Healthy older adults, Kinematic, Reference values, Spatiotemporal

## Abstract

Objective: This preliminary study aimed to explore the reference values of spatiotemporal and kinematic parameters in the lower extremities and trunk during gait for the healthy older adults. Methods: Walking speed, stride length and time, cadence, walk ratio, and step width were calculated as spatiotemporal parameters of gait. Forward tilting of the trunk (FTT), hip flexion and extension, knee flexion and extension, and their laterality were measured as peak angles during one-gait cycle. The bootstrap method was conducted to estimate the 95% confidence interval (CI). Results: This study included 334 healthy older adults (255 women). The following gait parameters were estimated with 95%CI: walking speed (95%CI 1.21–1.30), cadence (95%CI 116.35–121.20), walk ratio (95%CI 0.0055–0.0060), step width (95%CI 0.15–0.17), FTT (95%CI 1.91–4.19), hip flexion (95%CI 28.54–31.01), hip extension (95%CI 19.30–22.27), knee extension (95%CI 0.09–0.14), laterality of hip flexion (95%CI 1.31–2.02), laterality of hip extension (95%CI 1.32–1.97), laterality of knee flexion (95%CI 3.41–4.77), and laterality of knee extension (95%CI 0.07–0.13) in men, and walking speed (95%CI 1.28–1.34), walk ratio (95%CI 0.0050–0.0054), FTT (95%CI 2.54–3.73), hip flexion (95%CI 32.80–34.28), laterality of hip flexion (95%CI 1.65–2.05), laterality of hip extension (95%CI 2.06–2.57), and laterality of knee flexion (95%CI 3.04–3.89) in women. Conclusion: This study suggested provisional reference values of spatiotemporal and kinematic parameters in the lower extremities and trunk during gait for the healthy older adults.

**W**alking speed has been outlined as a useful clinical indicator of health status in the older adults^1)^ and a predictor of mortality and falls.^2)^ Older adults at risk of falls exhibit negative changes in the gait spatiotemporal parameters, such as reduced gait speed, decreased stride length, and increased variability of stride times.^3^^–^^5)^ Furthermore, the gait kinematics can predict mobility disorders in the older adults.^6)^

According to the National Cancer Institute at the National Institutes of Health, the reference intervals are values used by a physician to interpret the patients’ test results.^7)^ In spatiotemporal parameters of gait, the reference intervals for the older adults are known to be influenced by sex, age, and height.^8^^–^^10)^ Moreover, the sex-specific differences exist in the gait kinematic parameters.^11^^,^^12)^

In the gait kinematic parameters, although the relationship of the trunk angle to the pelvis during gait is related to age,^13)^ no reference intervals are available for the gait kinematics of the healthy older adults who are not frail. New sensors introduced into the clinical settings, such as the Kinect sensor, make it possible to easily obtain the coordinate data on gait.^14)^ The development of a reference range for kinematics using such technology could be one method of an early and reliable detection of health problems. Therefore, this study aimed to estimate reference data on spatiotemporal and kinematic parameters in the lower extremities and trunk during gait for the healthy older adults. If the reference intervals are estimated, the measurements of gait will provide a useful clinical information for an early detection of gait impairments.

## Methods

### Study design and population

This was a cross-sectional, observational study. Participants were recruited in the community centers and gymnasiums in Hiroshima Prefecture, Japan, from November 2020 to December 2020. Participants were included if they met the following criteria: (1) community-dwelling individuals aged 65 yrs or over and (2) individuals with an independent mobility. The following were the exclusion criteria: (1) suspected cognitive impairment and (2) serious illness (unstable cardiovascular disease, severe respiratory impairment, stroke, diabetic peripheral neuropathy, Parkinson’s disease, or rheumatoid/arthritis). Before the study enrollment, participants were informed about the purpose of the study and signed a consent form. This study was conducted according to the Declaration of Helsinki and approved by the institutional review board of Hiroshima University (No. 02–05).

### Frailty

To estimate the reference intervals for the gait parameter in the healthy older adults, we examined the frailty status of unhealthy individuals. We evaluated frailty status using a revised Japanese version of the Cardiovascular Health Study criteria.^15)^

### Measurements

Measurements were conducted in a gym or meeting room of a public facility. The Microsoft Kinect V2 sensor (Microsoft Corporation, Redmond, WA, USA) was used as a markerless motion capture system. Data obtained from measurements were processed using Mobile Motion Visualizer AKIRA (MMV AKIRA, System Friend Inc., Hiroshima, Japan). Data reliability and validity obtained using this device and software were confirmed.^16)^

The Kinect sensor was placed opposite to the participant and 1 m away from the end of a 5-m walking path. The participants were then instructed to walk on the 5-m path once at their usual walking speed. In contrast to a previous study,^16)^ the current study established no criteria for clothing and conducted measurements with footwear on. One gait cycle on each side was extracted for analysis.

Using MMV AKIRA, we measured walking speed, stride length and time, and cadence as the spatiotemporal variables of gait. The walk ratio was calculated as the step length divided by cadence. The angles to be measured were forward tilting of the trunk (FTT), hip flexion and extension, and knee flexion and extension. The angle projected on the sagittal plane was analyzed. In the analysis, we used the left and right averages for spatiotemporal variables and each angle. Data processing has been described in detail in our previous study.^16)^

### Statistical analysis

Healthy older adults were defined as those not diagnosed with frailty. To develop a reference interval, a normality test was conducted for the physical fitness measurement data of the healthy older adults; a bootstrap method was used to estimate the 95% confidence interval (CI) if the distribution was normal.

The bootstrap method can estimate the population even if the original data at the time of sampling are small.^17)^ For those items whose gait parameter results were not normally distributed, a Box–Cox transformation was conducted, and the normality test was re-conducted. When the results were transformed to a normal distribution, the bootstrap method was used to estimate the 95% CI.

**Fig. 1.**
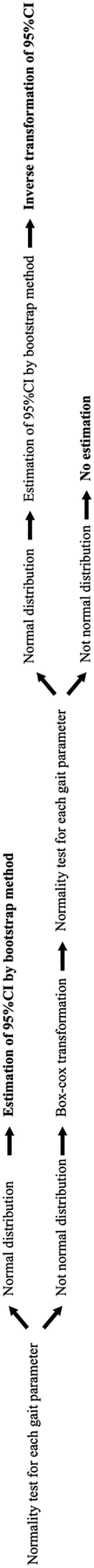
Inverse transformation of 95% CI

An inverse transformation was performed (Fig. 1). The normality test was performed using the Shapiro–Wilk test. The formula for the Box–Cox transformation was *x*’ = (*x*^λ^ − 1)/λ, and the formula for the inverse transformation was *x* = (*x*’ × λ + 1)^1/λ^.^18)^ In this case, *x* = log. The sample estimation by the bootstrap method was 1000 for each group.

CI, confidence interval

Finally, we calculated the 20th percentile value for each parameter after the reference intervals were calculated. Percentile values were calculated by sorting the values from smallest to largest. Calculations were performed to determine the position of each parameter within the reference interval for clinical use.

Outliers were handled using Huber’s M-estimation. We used JMP Pro 16 (SAS Inc., NC USA) for statistical analysis.

## Results

**Fig. 2.**
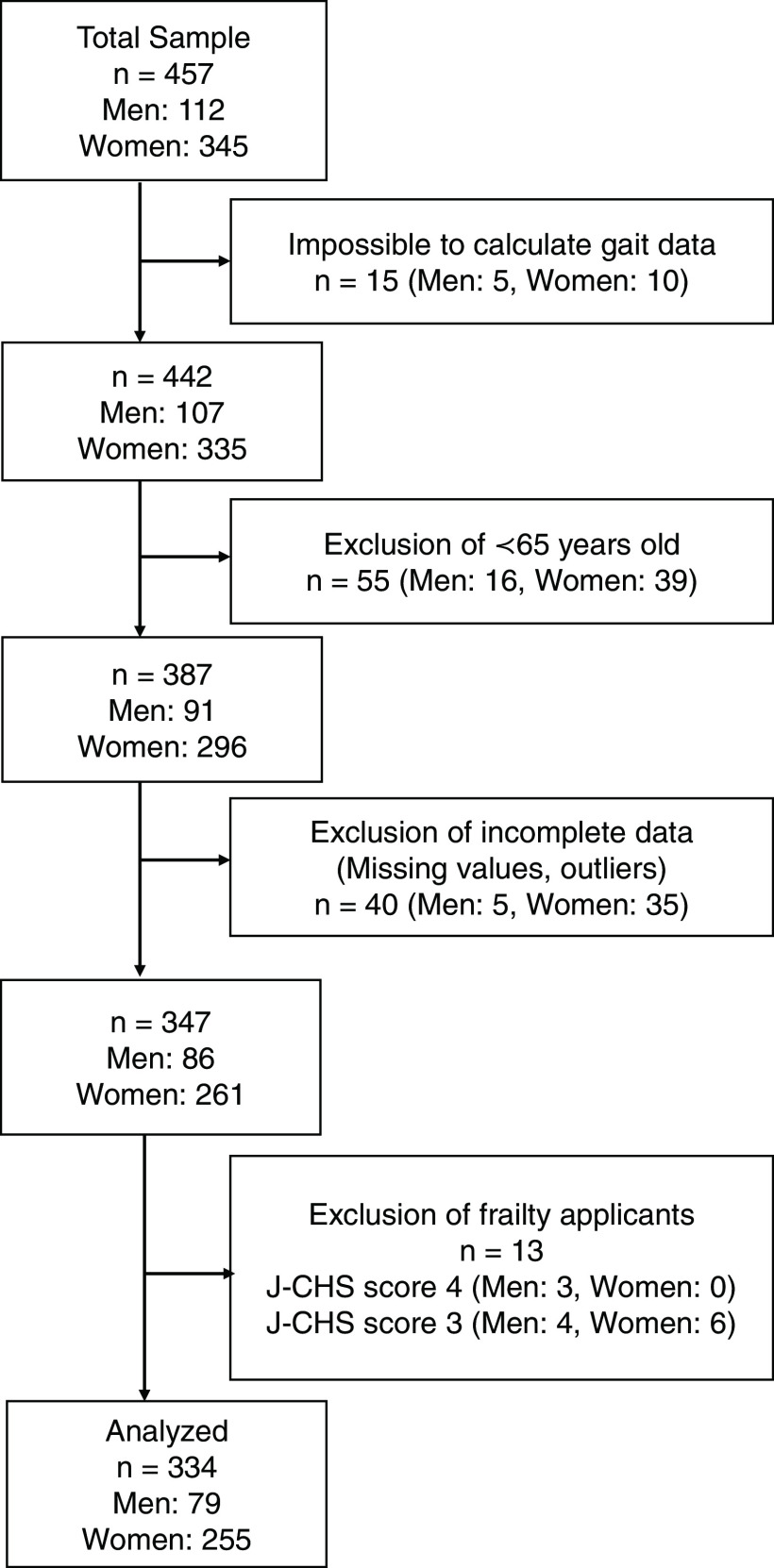
Flow diagram of the subject recruitment

A total of 334 participants were analyzed (Fig. 2). Participants wore a variety of clothing material, with some wearing wide-leg trousers that could easily be a source of error.^19)^ Table 1 presents the background and gait parameters of the participants. The normality test results showed normal distributions of walking speed, cadence, walk ratio, FTT, hip flexion, and hip extension in men and FTT and hip flexion in women.

Outliers were determined by Huber's M estimation. Those with a distance from the center greater than a factor of 4 were considered outliers. Subjects with outliers in any one of the kinematic parameters were excluded.

J-CHS, Japanese version of the Cardiovascular Health Study criteria

**Table 1.** Background and gait parameters of participants

	Men, n = 79	Women, n = 255
Age, years	76.63 (6.19)	73.97 (5.93)
Height, cm	164.15 (6.32)	152.93 (5.66)
Weight, kg	65.63 (9.47)	57.47 (11.95)
Walking speed, m/sec	1.26 (0.17)	1.29 (0.23)
Stride length, m	1.34 (0.18)	1.26 (0.20)
Stride time, sec	1.01 (0.11)	0.93 (0.12)
Cadence, step/min	118.74 (10.27)	125.00 (11.96)
Walk ratio	0.0057 (0.0009)	0.0051 (0.0008)
Step width, m	0.16 (0.06)	0.14 (0.05)
Forward tilting of the trunk, deg	3.16 (5.21)	3.12 (4.76)
Hip flexion, deg	29.69 (5.44)	33.55 (5.89)
Hip extension, deg	20.79 (6.36)	21.52 (6.64)
Knee flexion, deg	43.87 (5.00)	46.12 (5.91)
Knee extension, deg	0.17 (0.18)	0.62 (0.88)

Outliers are handled using Huber's m-estimation. Mean (SD), SD, standard deviation

The Box–Cox transformation was performed for the gait parameters that did not show a normal distribution, and the normality test was re-conducted. Consequently, the following gait parameters were normally distributed: step width; knee extension; and laterality of hip flexion, hip extension, knee flexion, and knee extension in men and walking speed; walk ratio; and laterality of hip flexion, hip extension, and knee flexion in women.

**Fig. 3.**
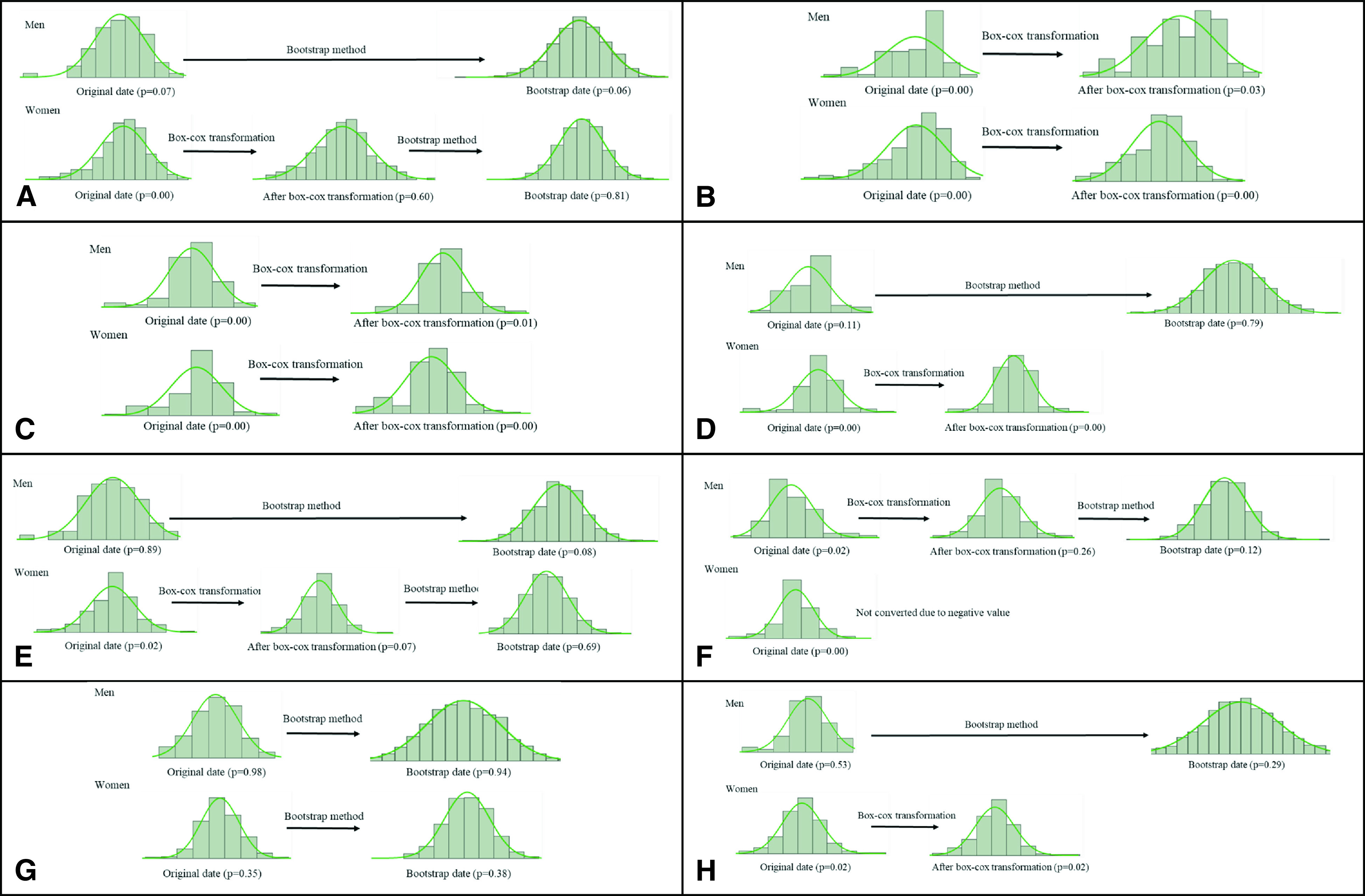

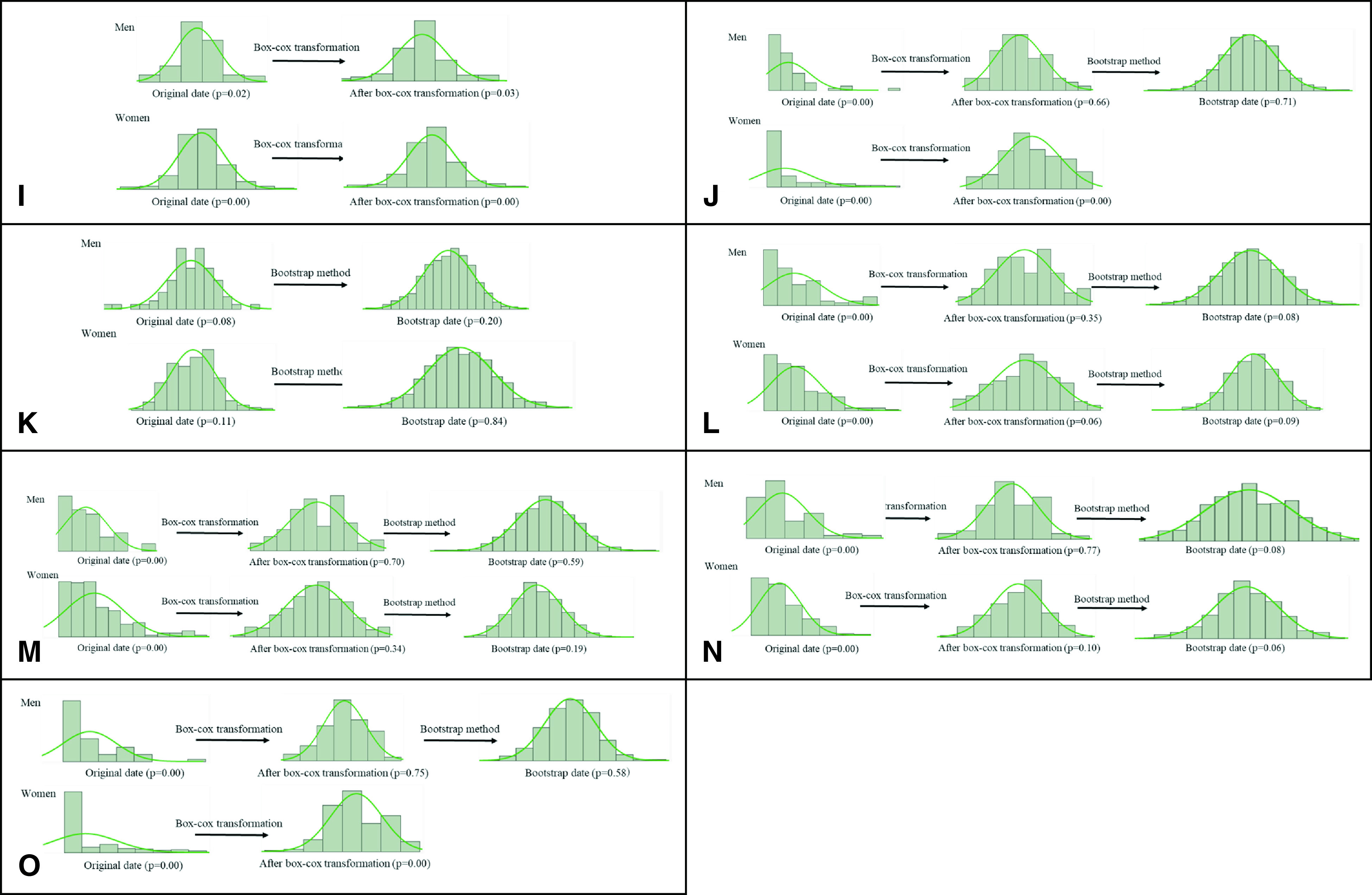
The reference intervals for the normally distributed gait parameters

Using the bootstrap method, the reference intervals for the normally distributed gait parameters were estimated (Fig. 3). This method was used to estimate the reference intervals for the following gait parameters: walking speed; cadence; step width; FTT; hip flexion; hip extension; knee extension; and laterality of hip flexion, hip extension, knee flexion, and knee extension in men and walking speed; FTT; hip flexion; walk ratio; and laterality of hip flexion, hip extension, and knee flexion in women (Table 2). Furthermore, the values of the abovementioned gait parameters estimated by the bootstrap method were calculated for the 20th percentile intervals for men and women (Tables 3–5).

(**A**) Gait speed, m/sec; (**B**) Stride length, m; (**C**) Stride time, sec; (**D**) Cadence, step/min; (**E**) Walk ratio; (**F**) Step width, m; (**G**) Forward tilting of the trunk, deg; (**H**) Hip flexion, deg; (**I**) Hip extension, deg; (**J**) Knee flexion, deg; (**K**) Knee extension, deg; (**L**) Laterality of hip flexion, deg; (**M**) Laterality of hip extension, deg; (**N**) Laterality of knee flexion, deg; (**O**) Laterality of knee extension, deg

**Table 2.** Reference interval for each gait parameter

	Men	Women
Spatiotemporal parameters		
Walking speed, m/sec	1.21–1.30*	1.28–1.34**
Stride length, m	N/A	N/A
Stride time, sec	N/A	N/A
Cadence, step/min	116.35–121.20*	N/A
Walk ratio	0.0055–0.0060*	0.0050–0.0054**
Step width, m	0.15–0.17**	N/A
Kinematics parameters		
Forward tilting of the trunk, deg	1.91–4.19*	2.54–3.73*
Hip flexion, deg	28.54–31.01*	32.80–34.28*
Hip extension, deg	19.30–22.27*	N/A
Knee flexion, deg	N/A	N/A
Knee extension, deg	0.09–0.14**	N/A
Kinematics parameters (laterality)		
Laterality of hip flexion, deg	1.31–2.02**	1.65–2.05**
Laterality of hip extension, deg	1.32–1.97**	2.06–2.57**
Laterality of knee flexion, deg	3.41–4.77**	3.04–3.89**
Laterality of knee extension, deg	0.07–0.13**	N/A

*: Raw data were normally distributed and estimated with 95% CI using the Bootstrap method

**: Normal distribution was observed using the Box-cox transformation after the Bootstrap method and then 95%CI were estimated

N/A: Not applicable (raw data were not normally distributed after the Box-cox transformation); CI, confidence interval

**Table 3.** 20% value intervals of the spatiotemporal parameters

	Men	Women
Walking speed, m/sec		
80%–	>1.28	>1.33
60–80%	>1.27, ≤1.28	>1.32, ≤1.33
40–60%	>1.26, ≤1.27	>1.31, ≤1.32
20–40%	>1.25, ≤1.26	>1.30, ≤1.31
–20%	≤1.25	≤1.30
Stride length, m		
80%–	N/A	N/A
60–80%	N/A	N/A
40–60%	N/A	N/A
20–40%	N/A	N/A
–20%	N/A	N/A
Stride time, sec		
80%–	N/A	N/A
60–80%	N/A	N/A
40–60%	N/A	N/A
20–40%	N/A	N/A
–20%	N/A	N/A
Cadence, step/min		
80%–	>119.72	N/A
60–80%	>119.03, ≤119.72	N/A
40–60%	>118.36, ≤119.03	N/A
20–40%	>117.68, ≤118.36	N/A
–20%	≤117.68	N/A
Walk ratio		
80%–	>0.00583	>0.00519
60–80%	>0.00577, ≤0.00583	>0.00517, ≤0.00519
40–60%	>0.00572, ≤0.00577	>0.00514, ≤0.00517
20–40%	>0.00566, ≤0.00572	>0.00511, ≤0.00514
–20%	≤0.00566	≤0.00511
Step width, m		
80%–	>0.163	N/A
60–80%	>0.160, ≤0.163	N/A
40–60%	>0.156, ≤0.160	N/A
20–40%	>0.153, ≤0.156	N/A
–20%	≤0.153	N/A

N/A: Not applicable (raw data were not normally distributed after the Box-cox transformation)

**Table 4.** 20% value intervals of kinematic parameters

	Men	Women
Forward tilting of the trunk, deg		
80%–	>3.66	>3.36
60–80%	>3.33, ≤3.66	>3.20, ≤3.36
40–60%	>3.03, ≤3.33	>3.04, ≤3.20
20–40%	>2.27, ≤3.03	>2.87, ≤3.04
–20%	≤2.27	≤2.87
Hip flexion, deg		
80%–	>30.22	>33.88
60–80%	>29.87, ≤30.22	>33.66, ≤33.88
40–60%	>29.54, ≤29.87	>33.46, ≤33.66
20–40%	>29.22, ≤29.54	>33.24, ≤33.46
–20%	≤29.22	≤33.24
Hip extension, deg		
80%–	>21.40	N/A
60–80%	>20.95, ≤21.40	N/A
40–60%	>20.63, ≤20.95	N/A
20–40%	>20.23, ≤20.63	N/A
–20%	≤20.23	N/A
Knee flexion, deg		
80%–	N/A	N/A
60–80%	N/A	N/A
40–60%	N/A	N/A
20–40%	N/A	N/A
–20%	N/A	N/A
Knee extension, deg		
80%–	>0.121	N/A
60–80%	>0.114, ≤0.121	N/A
40–60%	>0.108, ≤0.114	N/A
20–40%	>0.102, ≤0.108	N/A
–20%	≤0.102	N/A

N/A: Not applicable (raw were not normally distributed after the Box-cox transformation)

**Table 5.** 20% value intervals of laterality of kinematic parameters

	Men	Women
Laterality of hip flexion, deg		
80%–	>1.79	>1.94
60%–80%	>1.67, ≤1.79	>1.88, ≤1.94
40%–60%	>1.57, ≤1.67	>1.82, ≤1.88
20%–40%	>1.48, ≤1.57	>1.76, ≤1.82
–20%	≤1.48	≤1.76
Laterality of hip extension, deg		
80%–	>1.76	>2.40
60%–80%	>1.75, ≤1.66	>2.32, ≤2.40
40%–60%	>1.58, ≤1.66	>2.25, ≤2.32
20%–40%	>1.49, ≤1.58	>2.18, ≤2.25
–20%	≤1.49	≤2.18
Laterality of knee flexion, deg		
80%–	>4.40	>3.61
60%–80%	>4.16, ≤4.40	>3.49, ≤3.62
40%–60%	>3.99, ≤4.16	>3.39, ≤3.49
20%–40%	>3.80, ≤3.99	>3.29, ≤3.39
–20%	≤3.80	≤3.29
Laterality of knee extension, deg		
80%–	>0.11	N/A
60%–80%	>0.10, ≤0.11	N/A
40%–60%	>0.09, ≤0.10	N/A
20%–40%	>0.08, ≤0.09	N/A
–20%	≤0.08	N/A

N/A: Not applicable (raw were not normally distributed after the Box-cox transformation)

## Discussion

This study showed a reference interval defined as the 95% region, with 20, 40, 60, and 80th percentiles in the spatiotemporal and kinematic parameters during gait for the older adults aged 65 yrs or over grouped according to sex.

The reference intervals of spatiotemporal gait parameters may be a useful metrics for gait parameters in the older adults.^8^^,^^9)^ Conversely, previous studies investigated kinematic parameters during gait for the older adults and found a significant sex and age difference.^11^^,^^12)^ However, they did not estimate the reference intervals of the kinematic parameters for individuals aged 65 yrs or older or suggest an index for detecting the early stages of abnormalities in kinematic gait parameters.^11^^,^^12)^ Moreover, a previous study reported a strong association between age and the trunk angle in relation to the pelvis during gait.^13)^ In contrast to the previous studies, the present study is the first to estimate the reference intervals for both spatiotemporal and kinematic parameters in the lower extremities and trunk during gait in the older adults. Although the participants of the abovementioned studies were independent in their activities of daily living, our study included only relatively healthy older adults, except for frail individuals. Therefore, our research follows the principle that reference values should be developed based on the results of the healthy individuals,^7)^ which is a strength of this study.

Individuals with Alzheimer’s disease had shorter stride lengths than the healthy adults (<0.50 m).^20)^ Conversely, older adults without a history of falls showed a higher cadence (120 ± 7 steps/min) than individuals with a history of falls within the previous 6 months,^21)^ which is within the range of reference intervals in the present study. Moreover, our findings are consistent with those reported in a previous study on community-dwelling older Japanese adults, who demonstrated a walking speed of 1.26 and 1.27 m/s and a stride length of 1.21 and 1.15 m in men and women, respectively.^9)^ Therefore, reference intervals from the lower 20% to the upper 80% of the spatiotemporal parameters for the healthy older adults in the present study can provide information for detecting the early health disorders, including falls and cognitive impairments.

This study is novel because the reference intervals for gait in the older adults were estimated based on the kinematics and spatiotemporal parameters. The present study found that the reduced peak hip extension angle (11.1°) during gait in older fallers negatively contributed to the stride length and walking speed^21)^ and was lower than the hip extension angle (men, 20.79°; women, 21.52°). Moreover, our findings showed an FTT (men, 3.16°; women, 3.12°) that differed from the increased FTT (4.28°) during gait observed in patients with stroke.^22)^ Therefore, compared to those reported in the previous studies, our gait kinematics data presented the gait characteristics of the healthy older adults and may be helpful in monitoring the gait movement of the healthy older adults.

The current study had some limitations. First, data from only one gait cycle was used in our analysis, which may not represent the gait features of the participants and may affect the robustness of our findings. Second, we observed errors in the lower limb joint angles, which cannot be disregarded clinically.^16)^ Moreover, no clothing criteria were established, which might have affected the measurement values. The spatiotemporal parameters based on knee joint coordinates have been reported to have errors ranging 2.6%–4.4% for typical trousers and 3.4%–7.7% for wide-leg trousers and skirts.^19)^ which may affect the indices related to the knee joint coordinates. Third, errors could exist between the statistical distributions estimated by the resampling technique using the bootstrap method and the actual distributions. Fourth, the kinematic parameters of the ankle joint could not be correctly obtained using the Kinect system. Therefore, our estimations based on the markerless motion capture system could differ from the reference values calculated by the measured values in a more rigorous environment.

Despite these limitations, our results have potentially significant implications. Regarding the reference intervals in this study, our findings may estimate to what extent the gait ability is improved by an exercise training in the older adults. In addition, the reference intervals for the laterality of each peak joint angle may help to evaluate gait disorders considering reports of gait asymmetry in diseases, such as diabetes and peripheral neuropathy, in which the disorder presents on one side.^23)^

## Conclusions

Our study estimated the reference data for spatiotemporal and kinematic health parameters in the lower extremities and trunk during gait for the healthy older adults.

## Acknowledgments

This study was supported by a grant from Systemfriend Inc. (Hiroshima, Japan) and Chugoku Regional Innovation Research Center (Hiroshima, Japan).

## Conflicts of Interest

The authors declare no conflicts of interest.
